# AMPK-Directed Therapeutics for MASLD: Mechanistic Rationale, Activator Classes, and Clinical Translation

**DOI:** 10.3390/ijms27188358

**Published:** 2026-09-19

**Authors:** Reeju Amatya, Ji Yeon Hyun, Jun Hak Lee, Kyoung Ah Min, Meong Cheol Shin

**Affiliations:** 1 College of Pharmacy and Research Institute of Pharmaceutical Sciences, Gyeongsang National University, 501 Jinju Daero, Jinju 52828, Gyeongnam, Republic of Korea; 2 Phytoeco, Inc., Bldg. B, Room 404, 197 Injero, Gimhae 50834, Gyeongnam, Republic of Korea; phytoeco@naver.com; 3 Wasong Agricultural Co., Ltd., Bldg. B, Room 403, 197 Injero, Gimhae 50834, Gyeongnam, Republic of Korea; 4 College of Pharmacy and Inje Institute of Pharmaceutical Sciences and Research, Inje University, 197 Injero, Gimhae 50834, Gyeongnam, Republic of Korea; dlwnsgkr2341@gmail.com

**Keywords:** AMP-activated protein kinase (AMPK), metabolic-associated steatotic liver disease (MASLD), hepatic lipid metabolism, allosteric activation, isoform selectivity, direct AMPK activators

## Abstract

Metabolic-associated steatotic liver disease (MASLD) affects an estimated 25–30% of the global adult population and represents a leading cause of liver-related morbidity. However, approved pharmacological therapies remain strictly limited. AMP-activated protein kinase (AMPK), a highly conserved heterotrimeric serine/threonine kinase and major regulator of cellular energy homeostasis, is chronically suppressed in the steatotic liver through hyperinsulinemia, ceramide signaling, oxidative stress, and epigenetic silencing of its upstream activator LKB1. This suppression removes a critical brake on de novo lipogenesis while simultaneously impairing fatty acid β-oxidation, mitochondrial quality control, anti-inflammatory signaling, and hepatic stellate cell quiescence. Thus, AMPK restoration may serve as an effective strategy capable of addressing multiple pathological drivers of MASLD. This review examines the molecular architecture of the AMPK heterotrimer, the downstream signaling networks governing hepatic lipid metabolism, glucose homeostasis, and fibrogenesis, and a spectrum of AMPK-activating pharmacology relevant to MASLD. Furthermore, it would introduce indirect activators including metformin, berberine, resveratrol, and related phytochemicals, as well as direct allosteric activators targeting the ADaM site, such as A-769662, PF-06409577, MK-8722, Compound 991, and the clinical-stage compound O304. Translational challenges confronting this therapeutic class are critically evaluated.

## 1. Introduction

Metabolic dysfunction-associated steatotic liver disease (MASLD) is now the most common chronic liver disease worldwide, affecting more than a third of the global adult population [1,2], and is a major cause of cirrhosis, hepatocellular carcinoma, and liver-related mortality [3]. It is closely linked to obesity, insulin resistance, type 2 diabetes mellitus, dyslipidemia, and cardiovascular disease, reflecting its nature as both a hepatic and systemic metabolic disorder [4]. Disease progression is driven by the convergence of nutrient excess, mitochondrial dysfunction, oxidative stress, chronic inflammation, and maladaptive tissue repair, which together promote transition from simple steatosis to metabolic dysfunction-associated steatohepatitis (MASH), fibrosis, and end-stage liver disease [5,6]. Despite its growing clinical burden, approved disease-modifying pharmacotherapy remains limited: resmetirom (Rezdiffra^®^), a thyroid hormone receptor-β agonist, received accelerated US Food and Drug Administration (FDA) approval in March 2024 as the first licensed treatment for MASH, but its indication is restricted to non-cirrhotic disease with moderate-to-advanced hepatic fibrosis (stages F2–F3), leaving the broader MASLD population without approved pharmacological options [7,8]. Moreover, resmetirom’s clinical utility is tempered by a tolerability profile dominated by diarrhea and nausea, a risk of hepatotoxicity that necessitates periodic liver-enzyme monitoring, and a substantial treatment cost—estimated at roughly $50,000–67,000 per patient over the treatment period—that has yielded mixed cost-effectiveness conclusions across independent analyses [9,10]. These practical constraints, together with its narrow non-cirrhotic F2-F3 indication, leave most of the MASLD spectrum without an approved option and reinforce the rationale for pursuing mechanistically distinct, more broadly applicable strategies such as AMP-activated protein kinase (AMPK) activation.

AMPK has emerged as a particularly compelling therapeutic target in this context. AMPK is an evolutionarily conserved serine/threonine kinase that functions as a central sensor of cellular energy stress, coupling changes in the intracellular AMP:ADP:ATP ratio to coordinated metabolic responses [11]. In the liver, AMPK directly opposes the major metabolic abnormalities of MASLD by inhibiting de novo lipogenesis (principally through phosphorylation and inactivation of acetyl-CoA carboxylase (ACC)) and by promoting fatty acid β-oxidation, mitochondrial function, autophagy, and metabolic flexibility [12,13]. Beyond these metabolic effects, AMPK also suppresses inflammatory and fibrogenic signaling, extending its therapeutic relevance across multiple stages of disease progression [14,15].

The importance of this pathway is reinforced by the consistent observation that hepatic AMPK activity is chronically suppressed in MASLD [16]. Hyperinsulinemia, oxidative stress, mitochondrial dysfunction, and inflammatory signaling converge to impair AMPK activation in the steatotic liver [16,17]. Ceramide accumulation contributes indirectly to this suppression: elevated intrahepatic ceramides activate the NLRP3 inflammasome, triggering pro-inflammatory cytokine release that further inhibits AMPK activity and sustains a feedforward cycle of lipotoxicity and metabolic dysfunction [18]. Together, these mechanisms disable a major endogenous defense against metabolic overload, and restoration of AMPK activity has consequently become a mechanistically coherent strategy for simultaneously targeting steatosis, inflammation, and fibrosis [14,15].

Progress in this field has been accelerated by advances in the structural biology of AMPK. The kinase functions as an obligate heterotrimer composed of a catalytic α-subunit and regulatory β- and γ-subunits. Multiple isoforms exist for each subunit (α1, α2; β1, β2; γ1, γ2, γ3), permitting the assembly of 12 distinct αβγ combinations that differ in tissue distribution, subcellular localization, and pharmacological responsiveness [19]. AMP binding to the γ-subunit activates the complex by three complementary mechanisms: promotion of Thr172 phosphorylation by the upstream kinase LKB1, inhibition of Thr172 dephosphorylation by protein phosphatases, and direct allosteric activation—effects collectively opposed by ATP binding. Calcium/calmodulin-dependent protein kinase kinase β (CaMKKβ) phosphorylates the same Thr172 residue independently of adenine nucleotide status, in response to elevations in intracellular Ca^2+^ [11]. These structural insights have enabled the development of direct allosteric activators targeting defined sites within the AMPK complex, alongside continued investigation of indirect activators such as metformin and berberine [12,14].

This review introduces AMPK as a therapeutic target in MASLD from structural, mechanistic, and translational perspectives. We first summarize the molecular architecture of the AMPK heterotrimer and the structural basis of its regulation. We then discuss the role of AMPK in hepatic metabolism and MASLD pathogenesis, review current pharmacological strategies for AMPK activation, and consider the major challenges that continue to limit clinical translation.

## 2. Molecular Architecture of the AMPK Heterotrimer

**Figure 1:**
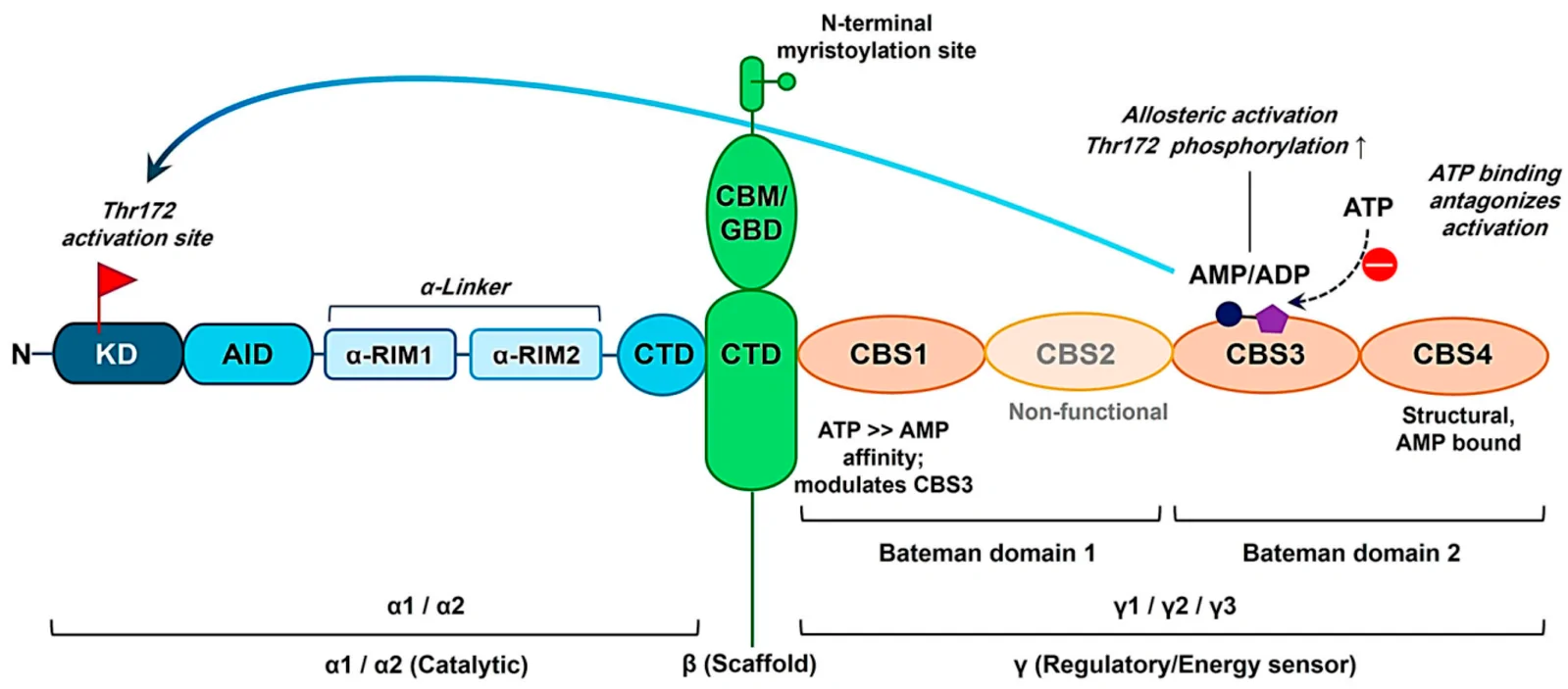
Domain architecture of the heterotrimeric AMPK complex (α/β/γ-subunits) and key regulatory sites. The catalytic α-subunit contains the kinase domain (KD) with the Thr172 activation site, an autoinhibitory domain (AID), an α-linker (α-RIM1/α-RIM2), and a C-terminal domain (CTD). The scaffold β-subunit contains the carbohydrate-binding module/glycogen-binding domain (CBM/GBD) and CTD, with a N-terminal myristoylation site mediating membrane/glycogen localization. The regulatory γ-subunit comprises four cystathionine-β-synthase (CBS) domains organized into two Bateman domains: Bateman domain 1 (CBS1–CBS2, where CBS1 shows high ATP-to-AMP binding affinity and modulates CBS3, while CBS2 is non-functional in most isoforms) and Bateman domain 2 (CBS3–CBS4, where CBS3 binds AMP/ADP to drive allosteric activation and promote Thr172 phosphorylation (indicated with an upward arrow), antagonized by ATP binding, while CBS4 is structurally bound to AMP constitutively).

AMPK is a highly conserved serine/threonine kinase that functions as the main regulator of cellular energy homeostasis [20]. The enzyme exists as an heterotrimeric complex comprising a catalytic α-subunit and two regulatory subunits, β and γ [21]. This trimeric architecture is essential not only for kinase activity but also for the allosteric regulation that enables AMPK to serve as a precise sensor of cellular energetic status [20,21]. Figure 1 illustrates the structure of the AMPK.

### 2.1. The Catalytic α-Subunit

The α-subunit constitutes the catalytic core of the AMPK complex and is expressed in mammals as two isoforms, α1 (encoded by PRKAA1) and α2 (encoded by PRKAA2) [21,22]. Although both isoforms share a common domain organization, they differ in their tissue distribution and subcellular localization: α2 is predominantly expressed in skeletal muscle, cardiac muscle, and liver, whereas α1 exhibits a broader, more ubiquitous expression profile [22,23]. Structurally, the α-subunit is organized into several critical functional domains. The N-terminal kinase domain (KD; approximately residues 11–267) harbors the ATP-binding site and the activation loop containing the critical Thr172 phosphorylation site (Thr174 in the α1 isoform; conventionally designated Thr172 after its position in rat/human α2), phosphorylation of which is required for full catalytic activation and confers an approximately 100-fold increase in kinase activity [23,24]. Immediately C-terminal to the KD lies the autoinhibitory domain (AID; residues ∼313–392), which folds back onto the kinase domain to suppress basal activity in the absence of upstream stimuli; release of this autoinhibition constitutes a key step in AMPK activation [25]. A flexible α-linker region (∼residues 395–487) connects the AID to the C-terminal domain (CTD) and contains two regulatory-subunit-interacting motifs (α-RIM1 and α-RIM2) that undergo conformational rearrangements upon AMP binding to the γ-subunit, thereby facilitating allosteric communication between subunits [21,25]. The CTD itself mediates interaction with the β-subunit, anchoring the α-subunit within the trimeric complex [21].

### 2.2. The Regulatory β-Subunit

The β-subunit functions as a molecular scaffold, bridging the α- and γ-subunits to maintain the structural integrity of the trimeric complex [21]. Two mammalian isoforms exist, β1 and β2 (encoded by PRKAB1 and PRKAB2, respectively), with β2 predominating in skeletal muscle and β1 in the liver [22,23]. Two functionally important domains characterize the β-subunit. The carbohydrate-binding module (CBM; also referred to as the glycogen-binding domain, GBD; residues ∼68–163) binds glycogen and other complex carbohydrates, an interaction thought to direct AMPK to glycogen particles and thereby position the kinase in proximity to substrates involved in glycogen synthesis, including glycogen synthase [21,26]. The unstructured N-terminal region of the β-subunit (preceding the CBM) is cotranslationally myristoylated at Gly2, a modification that facilitates membrane association and is required for AMP-stimulated Thr172 phosphorylation and maximal AMPK activation [21]. The C-terminal domain (CTD) of the β-subunit forms the central hub of the trimeric complex, engaging both α- and γ-subunits through well-defined hydrophobic and polar contacts; structural studies have established that the β-CTD is essential for complex stability and for transmitting conformational signals between the regulatory and catalytic components [21,25].

### 2.3. The Regulatory γ-Subunit

The γ-subunit serves as the primary energy-sensing component of the AMPK complex. Three mammalian isoforms are recognized (γ1, γ2, γ3), of which γ1 is ubiquitously expressed while γ2 and γ3 have tissue-restricted expression in cardiac and skeletal muscle, respectively [22,23]. The defining structural feature of the γ-subunit is the presence of four tandem cystathionine β-synthase (CBS) domains, which pair to form two Bateman domains [21,27]. These Bateman domains generate four potential nucleotide-binding sites (Sites 1–4), though Site 2 is non-functional owing to degenerate residues [21,25]. Of the remaining three functional sites, Site 3 (CBS3) is the primary allosteric regulatory site: AMP or ADP binding here promotes Thr172 phosphorylation by upstream kinases, protects against Thr172 dephosphorylation by protein phosphatases, and allosterically activates the phosphorylated enzyme, all three effects being antagonized by ATP [24,25,28]. Site 1 (CBS1) has an approximately 10-fold higher affinity for ATP than for AMP and, given that cellular ATP concentrations typically exceed AMP concentrations by up to 100-fold, is believed to be constitutively occupied by ATP in intact cells. Its role is to modulate the nucleotide-binding properties of the adjacent Site 3 rather than to serve as a dynamic AMP-sensing site [28,29]. Site 4 (CBS4) is constitutively occupied by AMP under most physiological conditions in a non-exchangeable manner, and contributes to structural stability and the fine-tuning of Site 3 sensitivity rather than to dynamic regulation [28,29]. The differential occupancy of these sites by AMP, ADP, or ATP allows the γ-subunit to act as a molecular rheostat, precisely calibrating AMPK activity in response to fluctuations in the cellular AMP/ATP and ADP/ATP ratios [20,25].

## 3. AMPK as a Central Metabolic Regulator in MASLD

**Figure 2:**
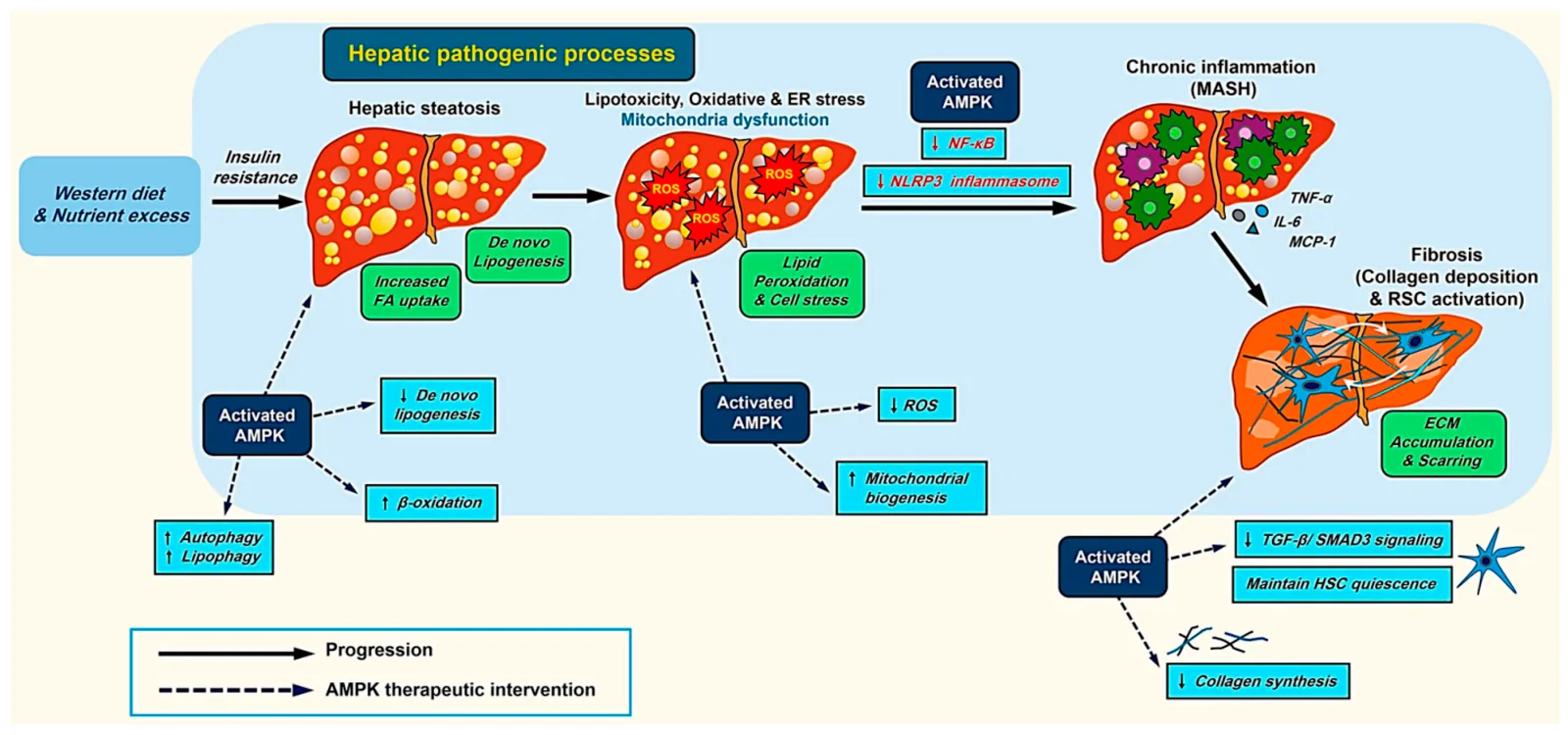
**AMPK-directed therapeutic intervention across MASLD pathogenesis.** Western diet and nutrient excess induce insulin resistance and hepatic steatosis via increased FA uptake and de novo lipogenesis; AMPK counters this by inhibiting ACC/SREBP1c and promoting β-oxidation and autophagy/lipophagy. Progressive lipotoxicity, oxidative/ER stress, and mitochondrial dysfunction drive ROS-mediated lipid peroxidation; AMPK reduces ROS and enhances mitochondrial biogenesis. Sustained stress activates NF-κB/NLRP3 signaling, producing chronic inflammation (MASH) with TNF-α, IL-6, and MCP-1 release; AMPK suppresses these inflammatory pathways. Chronic inflammation activates hepatic stellate cells (HSCs) and drives ECM accumulation and fibrosis; AMPK maintains HSC quiescence and reduces TGF-β/SMAD3 signaling and collagen synthesis. Solid arrows: disease progression; dashed arrows: AMPK-mediated intervention; upward arrows: increase; downward arrows: decrease.

AMPK functions as a major regulator of cellular energy homeostasis and plays a pivotal role in coordinating metabolic adaptation in hepatocytes [20,22]. Fluctuations in intracellular energy status (specifically increases in the AMP:ATP or ADP:ATP ratio during metabolic stress) promote adenine nucleotide binding to the γ-subunit, inducing conformational changes that facilitate phosphorylation of the α-subunit at Thr172 by upstream kinases [20,24]. Two principal upstream kinases mediate this phosphorylation: liver kinase B1 (LKB1), a constitutively active tumor suppressor that serves as the dominant AMPK kinase in the liver, and CaMKKβ, which activates AMPK in response to intracellular calcium signals [30,31,32]. Thr172 phosphorylation can increase AMPK activity by more than 100-fold, and under conditions of severe metabolic stress, where AMP-mediated allosteric activation combines with Thr172 phosphorylation, overall amplification can exceed 1000-fold above basal levels [20,22]. Inhibitory counter-regulation is exerted by Akt and S6K1, which phosphorylate the α1-subunit at Ser485 (α1)/Ser491 (α2), attenuating AMPK activity [33,34]. AMPK exerts multi-nodal control over MASLD pathogenesis, intervening at each stage of disease progression from hepatic steatosis through lipotoxicity/oxidative stress, chronic inflammation, and fibrosis (Figure 2).

### 3.1. Physiological and Pathological Triggers of AMPK Activation in the Liver

AMPK activation is elicited by a diverse array of physiological and pathological stimuli that share the common feature of either diminishing ATP production or augmenting ATP consumption [20,35]. Nutrient deprivation, encompassing both glucose withdrawal and amino acid deficiency, reduces glycolytic ATP output and impairs mitochondrial oxidative phosphorylation, respectively, each resulting in AMPK activation [20]. Hypoxia limits mitochondrial ATP synthesis by disrupting electron transport chain (ETC) function. AMPK activation under these conditions is further facilitated by LKB1 and is linked to the hypoxia-inducible factor (HIF) pathway [20,35]. During exercise and muscle contraction, repeated ATP hydrolysis by myosin ATPases rapidly depletes local ATP and elevates AMP, with both CaMKKβ (activated via Ca^2+^ release from the sarcoplasmic reticulum) and LKB1 contributing to AMPK activation in contracting muscle [20,22].

Mitochondrial dysfunction, including inhibition of ETC complex I by agents such as metformin, phenformin, or rotenone, impairs oxidative phosphorylation and reduces the ATP/AMP ratio, thereby activating AMPK [36]. Reactive oxygen species (ROS) can activate AMPK both by impairing mitochondrial function and through direct redox-dependent mechanisms [20]. A number of pharmacological agents also engage this pathway: AICAR (5-aminoimidazole-4-carboxamide ribonucleotide) is converted intracellularly to the AMP mimetic ZMP and activates AMPK without altering cellular ATP levels, while metformin and other biguanides act indirectly via complex I inhibition [35,36]. Finally, hormonal signals including leptin, adiponectin, and ghrelin activate AMPK in specific tissues, particularly the hypothalamus, where the kinase plays a well-established role in appetite regulation [22].

### 3.2. Downstream Signaling and Metabolic Coordination

Upon activation, AMPK works on a comprehensive metabolic reprogramming through the phosphorylation of a broad array of downstream substrates [20,22]. The logic governing these phosphorylation events is the simultaneous inhibition of anabolic, ATP-consuming processes and activation of catabolic, ATP-generating pathways, thereby restoring cellular energy balance [20].

#### 3.2.1. Lipid Metabolism

AMPK exerts profound and multilevel effects on lipid metabolism, inhibiting de novo lipogenesis and cholesterol biosynthesis while promoting fatty acid β-oxidation [37,38,39,40]. These effects are achieved through phosphorylation of acetyl-CoA carboxylase (ACC1/ACC2), HMG-CoA reductase (HMGCR), and the lipogenic transcription factors SREBP-1c and ChREBP, together with activation of PGC-1α; the specific phosphorylation sites and downstream consequences of each target are consolidated in Section 3.3.

#### 3.2.2. Glucose Homeostasis

AMPK promotes glucose uptake in skeletal muscle through phosphorylation of TBC1D1 and TBC1D4 (AS160), stimulating insulin-independent translocation of glucose transporter 4 (GLUT4) to the plasma membrane [41]. Glycolytic flux is enhanced through AMPK-mediated phosphorylation and activation of 6-phosphofructo-2-kinase (PFKFB3), which increases fructose-2,6-bisphosphate levels [20]. In the liver, AMPK reduces gluconeogenic gene expression (including PEPCK and G6Pase) by phosphorylating and inhibiting the CREB coactivator CRTC2 (TORC2) and the class IIa histone deacetylases HDAC4/5 [42]. Additionally, AMPK phosphorylates glycogen synthase to inhibit glycogen deposition, freeing glucose for immediate energetic utilization [20].

#### 3.2.3. Mitochondrial Biogenesis and Autophagy

AMPK promotes mitochondrial biogenesis through direct phosphorylation of PGC-1α (peroxisome proliferator-activated receptor gamma coactivator 1-alpha) at Thr177 and Ser538, and indirectly through SIRT1 activation, thereby stimulating oxidative capacity [40]. The kinase also inhibits mTORC1-driven anabolic signaling by phosphorylating TSC2 at Thr1227 and Ser1345 and by directly phosphorylating the mTORC1 component Raptor at Ser722 and Ser792 [43,44]. This mTORC1 inhibition indirectly promotes autophagy, facilitating the degradation and recycling of damaged organelles and macromolecules. AMPK additionally stimulates autophagy through direct phosphorylation of ULK1 (unc-51-like autophagy-activating kinase 1), a key initiator of the autophagic program [45]. Selective autophagy of dysfunctional mitochondria (mitophagy) is similarly promoted by AMPK-driven autophagic flux, contributing to mitochondrial quality control and attenuating ROS production [22,45].

### 3.3. Direct Metabolic Targets of AMPK in Hepatic Lipid Homeostasis

Within the liver, AMPK activation works on a coordinated shift from anabolic to catabolic metabolism that directly opposes the pathological lipid accumulation characteristic of MASLD [46,47]. Five direct substrates account for the majority of this action and are described here in full; they are referred back to, rather than re-explained, wherever individual AMPK-activating agents are discussed in Section 4. ACC exists as two isoforms: ACC1 (cytoplasmic) catalyzes the conversion of acetyl-CoA to malonyl-CoA for de novo fatty acid synthesis, while ACC2 (mitochondria-associated) generates malonyl-CoA that allosterically inhibits carnitine palmitoyltransferase-1 (CPT1), the rate-limiting enzyme for mitochondrial long-chain fatty acid import [37]. AMPK phosphorylates both isoforms at inhibitory serine residues (ACC1 Ser79; ACC2 Ser221 in humans, Ser212 in mice), simultaneously curtailing lipid synthesis and relieving CPT1 inhibition to enhance fatty acid β-oxidation [37]. At the transcriptional level, AMPK suppresses the lipogenic transcription factors SREBP-1c and ChREBP through direct phosphorylation and indirect inhibition of mTORC1-dependent processing, reducing expression of FASN, SCD1, ACC1, and other lipogenic genes [38,48]. AMPK additionally phosphorylates HMGCR at Ser872, inhibiting the rate-limiting step of cholesterol biosynthesis [39]. Finally, AMPK phosphorylates and activates PGC-1α at Thr177 and Ser538, enhancing its coactivation of PPARα to drive transcription of mitochondrial fatty acid oxidation genes and promote mitochondrial biogenesis [40]. Together, ACC, SREBP-1c/ChREBP, HMGCR, and PGC-1α constitute the principal direct metabolic targets through which AMPK opposes hepatic lipid accumulation; complementary suppression of gluconeogenesis via CRTC2 and HDAC4/5 and promotion of autophagic lipid catabolism (lipophagy) via ULK1 are addressed in Section 3.2.2 and Section 3.2.3, respectively [42,45,49].

### 3.4. AMPK Dysfunction in MASLD Pathogenesis

In MASLD and its inflammatory form MASH, hepatic AMPK activity is chronically suppressed through multiple converging mechanisms [47]. Chronic hyperinsulinemia, a hallmark of the underlying metabolic syndrome, drives Akt-mediated inhibitory phosphorylation of AMPKα at Ser485/491 introduced above (Section 3) while simultaneously activating SREBP-1c-driven lipogenesis detailed in Section 3.3, creating a metabolic state of impaired lipid catabolism alongside enhanced lipid synthesis [33,34]. Excess dietary saturated fatty acids, particularly palmitate, generate ceramide through the de novo ceramide synthesis pathway; ceramide activates protein phosphatase 2A (PP2A), which dephosphorylates and inactivates AMPK at Thr172 [50]. Mechanistically, ceramide can activate PP2A directly, by binding to the core AB′C heterotrimer in a manner that requires the regulatory B-subunit, thereby increasing the catalytic activity of the holoenzyme toward its phosphorylated substrates [51]; ceramide can additionally promote PP2A activation indirectly by displacing the endogenous inhibitor I2PP2A/SET, freeing the phosphatase for substrate engagement [52]. Mitochondrial dysfunction—a consistent pathological feature of MASLD—generates reactive oxygen species that oxidatively modify AMPK and its upstream kinases, further impairing energy sensing [20,47]. Gut-derived lipopolysaccharide, which reaches the liver in elevated concentrations due to increased intestinal permeability in MASLD, activates TLR4 signaling and NF-κB-dependent upregulation of inhibitory phosphatases [53]. At the epigenetic level, promoter hypermethylation of LKB1/STK11 and miR-33-mediated translational repression of AMPKα mRNA provide additional feedforward mechanisms sustaining AMPK suppression in the lipid-laden liver [54,55]. Collectively, these mechanisms ensure that the pathological conditions defining MASLD (lipid excess, insulin resistance, oxidative stress, and inflammation) conspire to disable the principal kinase responsible for restoring metabolic balance [47].

### 3.5. Anti-Inflammatory, Antifibrotic, and Therapeutic Implications

Beyond the Kupffer cell-centric perspective emphasized above, MASLD progression reflects a broader dysregulation of both innate and adaptive immunity within the liver and its associated adipose and intestinal compartments [56,57]. In addition to Kupffer cells, adipose tissue macrophages, neutrophils (including those generating neutrophil extracellular traps), monocyte-derived macrophages, dendritic cells, and innate lymphoid cell subsets (natural killer cells and ILC1-ILC3) collectively shape the hepatic inflammatory milieu that drives the transition from simple steatosis to steatohepatitis [57]. This innate inflammatory network is further modulated by adaptive immune cells: CD4^+^ and CD8^+^ T lymphocytes, Th17 cells, regulatory T cells (Tregs), natural killer T cells, mucosal-associated invariant T cells, and B lymphocytes infiltrate the MASLD liver and interact reciprocally with innate effectors, amplifying hepatocellular injury, promoting hepatic stellate cell activation, and, in advanced disease, contributing to the transition toward hepatocellular carcinoma [56,57]. The relative contribution of these immune compartments evolves with disease stage, and their crosstalk—rather than any single cell type in isolation—is now recognized as central to MASLD pathogenesis.

AMPK is expressed across this broader immune cell repertoire and exerts regulatory influence over both arms of immunity relevant to MASLD. In macrophages and dendritic cells, AMPK activation restrains NF-κB-dependent transcription of pro-inflammatory cytokines, promotes fatty acid oxidation over aerobic glycolysis, and favors acquisition of the anti-inflammatory M2-like phenotype, whereas loss of AMPKα1/β1 signaling sensitizes macrophages to LPS-driven M1 polarization and heightened pro-inflammatory cytokine output [58]. Analogous metabolic checkpoints operate in adaptive immunity: AMPK antagonizes mTORC1-driven glycolytic commitment in activated CD4^+^ T cells, thereby favoring fatty acid oxidation-dependent regulatory T cell differentiation over the glycolysis-dependent Th17 program, and pharmacological AMPK activators such as metformin and AICAR shift the Th17/Treg balance toward a regulatory, anti-inflammatory phenotype in preclinical models of chronic inflammatory disease [59]. In the context of MASLD, this dual innate–adaptive action suggests that AMPK-directed therapeutics may act not only on Kupffer cells and hepatic stellate cells, as detailed below, but also more broadly on the recruited macrophage, dendritic cell, and T-lymphocyte populations that sustain chronic low-grade hepatic inflammation, offering a mechanistic rationale for anti-inflammatory efficacy that extends beyond the resident macrophage compartment [58,59].

Beyond its metabolic functions, hepatic AMPK suppresses the inflammatory and fibrogenic processes that drive MASLD progression to MASH, cirrhosis, and hepatocellular carcinoma [47,60]. In hepatocytes and Kupffer cells, AMPK attenuates NF-κB signaling, suppresses NLRP3 inflammasome activation by reducing mitochondrial ROS and inhibiting mTORC1, and promotes autophagic clearance of damage-associated molecular patterns [60,61]. In hepatic stellate cells (HSCs), AMPK inhibits transdifferentiation to the profibrogenic myofibroblast phenotype through suppression of TGF-β/Smad3 signaling, mTORC1 inhibition, and promotion of lipid droplet autophagy that maintains HSC quiescence, collectively reducing collagen deposition and fibrosis progression [62]. AMPK also shapes extracellular matrix (ECM) turnover more directly: by restraining TGF-β/Smad3 signaling, AMPK activation limits the transcriptional induction of tissue inhibitor of metalloproteinases-1 (TIMP-1) that typically accompanies HSC activation, thereby favoring net matrix metalloproteinase (MMP)-mediated collagen degradation over deposition; pharmacological AMPK activation with the β1/β2 activator BI9774 reduced hepatic Timp1 transcript levels together with multiple collagen genes in a diet-induced MASH model [63], consistent with the AMPK-dependent reductions in fibrosis markers reported for the β1-selective activator PF-06409577 [64]. This relationship is nonetheless complex and context-dependent: HSC-intrinsic AMPK signaling has recently been shown to support the metabolic reprogramming required for HSC activation itself, with HSC-specific AMPKα deletion increasing hepatic MMP2/MMP9 activity and lowering TIMP-1 while attenuating fibrosis, in apparent contrast to the antifibrotic effects associated with hepatocyte or whole-body AMPK activation [65]. This discrepancy suggests that AMPK’s net effect on ECM turnover depends on the cell type and disease stage in which it is engaged, an important consideration for tissue-targeted AMPK-based therapeutics. These broad actions position AMPK as an attractive therapeutic target for MASLD across its full disease spectrum [47]. Pharmacological strategies to activate AMPK include indirect activators such as metformin (mitochondrial complex I inhibition) and exercise-mimetic compounds, and direct allosteric activators targeting the ADaM site (allosteric drug and metabolite binding site) at the α/β-subunit interface, including 991, MK-8722, and the clinical-stage compound O304 [25,36,66,67,68]. Cardiac glycogen accumulation observed with pan-AMPK activation has driven the development of liver-targeted or isoform-selective compounds to improve the therapeutic window [68]. The concurrent systemic benefits of AMPK activation-enhanced skeletal muscle glucose uptake and suppressed adipose lipolysis complement direct hepatic effects, reducing the metabolic substrate supply that fuels hepatic lipid accumulation [22,33]. Collectively, the metabolic, anti-inflammatory, and antifibrotic actions of AMPK, combined with advances in isoform-selective and tissue-directed pharmacology, support continued development of AMPK activators as a mechanistically compelling approach to MASLD therapy [47].

### 3.6. The Hepato-Splenic Feedback Loop: Portal Hypertension, Splenic Immune Activation, and AMPK-Directed Interruption

The mechanisms described above are not confined to the liver: chronic AMPK suppression sets off a self-reinforcing loop that extends along the hepato-splenic axis, linking intrahepatic injury to systemic immune activation [69,70,71]. The spleen was long treated as a passive bystander in liver disease, but a growing body of work positions it as an active contributor to disease progression [70]. As HSC-driven collagen deposition and sinusoidal capillarization (Section 3.5) distort hepatic architecture, intrahepatic vascular resistance climbs, producing sinusoidal and then portal hypertension. Because splenic blood drains into the liver via the shared portal circulation, this resistance is transmitted retrograde into the spleen, where it drives congestion and structural remodeling. Under these conditions, splenic macrophages become a source of TGF-β1 that is delivered to the liver through the portal vein and acts directly on HSCs—a route for spleen-to-liver fibrogenic signaling first described in rat models of splenic involvement in liver fibrosis [72,73]. In parallel, hepatokines and pro-inflammatory cytokines released by lipotoxic, NF-κB/NLRP3-activated hepatocytes and Kupffer cells (Section 3.5) reach the congested spleen through the same circulation and drive expansion of splenic macrophages and MDSCs, mirroring the MDSC accumulation seen in the obese, fatty liver [70,71]. Beyond cytokine exchange, the spleen also functions as a staging ground for cellular traffic into the liver. A splenic monocyte subset marked by CD11b^+^CD43hiLy6Clo differentiates locally before migrating to the fibrotic liver, where it converts into pro-fibrotic macrophages that activate HSCs and add to collagen deposition—a pathway demonstrated directly by splenectomy, splenocyte transfer, and lineage-tracing experiments in fibrotic mice [74]. Comparable trafficking of inflammatory monocytes and Th17 cells from spleen to liver has been proposed as a further route by which splenic activity sustains hepatic inflammation [70,74]. Taken together, this cellular and cytokine crosstalk means the spleen does not merely register liver injury passively; its output actively feeds back into the fibrotic process, coupling portal hemodynamics to systemic myeloid inflammation [70,74].

AMPK-directed therapeutics are positioned to interrupt this loop at more than one point, rather than acting solely on hepatocyte lipid metabolism. First, by restoring energy homeostasis in hepatocytes and HSCs, AMPK activators suppress de novo lipogenesis and HSC transdifferentiation (Section 3.3 and Section 3.5), limiting the structural contribution to intrahepatic vascular resistance. Second, AMPK activation in sinusoidal endothelial cells lowers portal pressure through a separate, more immediate mechanism: the AMPK agonist AICAR increases eNOS phosphorylation and nitric oxide production in these cells, producing acute sinusoidal vasodilation that reduces portal pressure in fibrotic rats without altering systemic hemodynamics [75]. By preserving sinusoidal architecture while also relieving portal pressure directly, AMPK activation should reduce splenic congestion and, with it, the downstream hyperactivation of splenic macrophages and MDSCs that otherwise supplies the liver with profibrogenic TGF-β1 [69,70,71]. Together, these hepatocyte-, HSC-, endothelial-, and immune-directed effects give AMPK-targeted pharmacology a mechanistic rationale for interrupting the hepato-splenic loop at both its vascular and immunological nodes—extending its relevance beyond the hepatocyte-centric framework of Section 3.1, Section 3.2, Section 3.3, Section 3.4 and Section 3.5.

## 4. Pharmacological Activation of AMPK

Pharmacological activators of AMPK can be broadly divided into two categories: (1) indirect activators, which stimulate AMPK through alterations in cellular energy status or upstream signaling pathways, and (2) direct activators, which interact with the AMPK complex itself to enhance kinase activity. Both of them have demonstrated beneficial metabolic effects in the treatment of MASLD and related metabolic diseases. Table 1 summarizes major pharmacological activators of AMPK that have demonstrated metabolic or therapeutic relevance in MASLD.

### 4.1. Indirect AMPK Activators

Indirect AMPK activators modulate the cellular energy state or upstream kinase pathways rather than engaging the AMPK complex directly. The majority of clinically used and naturally derived compounds in this class exert their effects through partial inhibition of mitochondrial oxidative phosphorylation, leading to reductions in ATP synthesis and consequent rises in intracellular AMP and ADP concentrations. Elevated AMP levels engage AMPK through dual mechanisms (allosteric activation of the catalytic subunit and protection of the Thr172 activation-loop phosphorylation from dephosphorylation by protein phosphatases) [20,104].

#### 4.1.1. Metformin

Metformin is a widely studied AMPK activator in metabolic diseases [105,106]. Metformin is a hydrophilic cationic compound that accumulates in hepatocytes following oral administration via organic cation transporter 1 (OCT1)-mediated uptake [107,108]. It reduces the efficiency of oxidative phosphorylation, lowering mitochondrial membrane potential and decreasing the rate of ATP synthesis. The decline in cellular ATP is accompanied by a proportional rise in AMP and ADP levels, shifting the AMP:ATP ratio toward values that favor AMPK activation [36,109]. AMPK activation by metformin engages the same hepatic metabolic targets detailed in Section 3.3, most notably ACC-mediated suppression of lipogenesis and stimulation of fatty acid β-oxidation together with SREBP-1c/ChREBP inhibition, collectively reducing intrahepatic fat content [37,76]. Recent mechanistic studies have also revealed that a significant component of metformin’s glucose-lowering effect in the liver works through AMPK-independent mechanisms. Specifically, inhibition of mitochondrial glycerophosphate dehydrogenase (mGPD) by metformin impairs lactate and glycerol conversion to glucose and reduces the hepatocyte redox state available to support gluconeogenesis [110,111]. Moreover, metformin has been shown to inhibit adenylate cyclase, which attenuates glucagon-stimulated cAMP production and suppresses gluconeogenic gene transcription [110]. A meta-analysis of clinical studies found that metformin reduced liver enzymes and body weight. However, it did not significantly improve hepatic inflammation or fibrosis scores compared to placebo [112,113].

#### 4.1.2. Berberine

Berberine is an isoquinoline alkaloid derived from medicinal plants such as Coptis chinensis and Berberis species. Similar to metformin, berberine activates AMPK indirectly through inhibition of mitochondrial oxidative phosphorylation, leading to reduced ATP levels and activation of the LKB1-AMPK signaling axis [78]. Berberine preferentially inhibits complex I of the mitochondrial respiratory chain at low micromolar concentrations, and the resulting increase in cellular AMP:ATP and ADP:ATP ratios provides the energetic signal that drives AMPK activation in hepatocytes [78,114]. In preclinical MASLD studies, berberine significantly reduced hepatic lipid accumulation, improved insulin sensitivity, and suppressed inflammatory signaling pathways, including nuclear factor-κB (NF-κB) and the NLRP3 inflammasome [115]. These effects reflect the ACC/SREBP-1c-centered mechanism already summarized in Table 1 and Section 3.3, evidenced transcriptionally by FASN downregulation and CPT1A/PPARA upregulation [115,116]. Notably, berberine has also been reported to modulate gut microbiota composition, which may contribute to reductions in hepatic inflammatory signaling through decreased portal vein endotoxin delivery [117,118]. A clinical trial comparing berberine with lifestyle intervention in patients with MASLD demonstrated that berberine produced comparable reductions in liver fat and metabolic parameters over a 16-week intervention period [118]. However, enhancing the oral bioavailability of berberine remains a challenge, due to its poor intestinal permeability, extensive P-glycoprotein-mediated efflux, and significant first-pass hepatic metabolism [78,119].

#### 4.1.3. Resveratrol

Resveratrol is a stilbenoid polyphenolic compound that has been well known for its metabolic regulatory and cardioprotective properties. The compound activates AMPK through mechanisms that involve both mitochondrial modulation and activation of the NAD^+^-dependent deacetylase SIRT1. Resveratrol competitively inhibits cAMP-degrading phosphodiesterases, elevating intracellular cAMP and activating Epac1, which in turn increases cytoplasmic Ca^2+^ levels and activates CaMKKβ-dependent AMPK phosphorylation [82]. Through the SIRT1-LKB1 signaling node, resveratrol also deacetylates and activates LKB1, enhancing its capacity to phosphorylate AMPK at Thr172 [120]. In preclinical studies, resveratrol reduced hepatic triglyceride accumulation, improved insulin sensitivity, attenuated inflammatory signaling, and promoted mitochondrial biogenesis through the AMPK-SIRT1-PGC-1α transcriptional cascade [121,122]. The AMPK-mediated phosphorylation of PGC-1α increased the expression of mitochondrial biogenesis genes and oxidative phosphorylation machinery, enhancing the hepatic capacity for fatty acid catabolism. Resveratrol also activated autophagy through AMPK-dependent inhibition of mTORC1, promoting lipophagy-mediated clearance of lipid droplets in steatotic hepatocytes [121]. Despite encouraging preclinical data, clinical studies with resveratrol have reported inconsistent results [123,124]. The primary limitation was its poor oral bioavailability, attributable to extensive intestinal and hepatic metabolism, and rapid systemic clearance [125].

This bioavailability limitation is mechanistic rather than merely a dosing shortfall: orally administered trans-resveratrol undergoes near-complete first-pass glucuronidation and sulfation in the intestinal epithelium and liver within minutes of absorption, yielding circulating concentrations of the pharmacologically active free (aglycone) form in the low nanomolar range even after gram-scale dosing several orders of magnitude below the concentrations required for AMPK activation in vitro and in the rat models that generated the encouraging preclinical data described above [125]. Because the unabsorbed and unmetabolized fraction reaches the colon, extensive biotransformation by the gut microbiota further compounds inter-individual variability in effective exposure. Clinical trials have also differed substantially in dose (500 mg to 3 g/day), formulation (unformulated crystalline resveratrol versus micronized preparations), duration (8 weeks to 6 months), and patient population (obese non-diabetic subjects versus biopsy-confirmed MASLD/MASH patients), factors that likely contributed to the divergent outcomes reported across studies; a meta-analysis of seven randomized controlled trials in MASLD patients found no significant benefit of resveratrol supplementation on hepatic or metabolic endpoints irrespective of dose or duration [126].

#### 4.1.4. Other Natural AMPK Activators

Epigallocatechin-3-gallate (EGCG), the most abundant catechin in green tea, activates AMPK in hepatocytes and other cell types through mechanisms involving mild mitochondrial uncoupling and activation of CaMKKβ, the latter stimulated by increased intracellular calcium signaling [127,128]. In diet-induced obese mice, green tea extract supplementation reduced hepatic steatosis by hepatic AMPK activation via the LKB1-AMPK pathway [85]. EGCG additionally promoted autophagy and autophagic flux in hepatocytes through AMPK–ULK1 signaling, facilitating intracellular lipid clearance [128,129]. Anti-inflammatory effects in the liver via AMPK-dependent attenuation of pro-inflammatory signaling have also been reported [130].

The flavonoids quercetin and kaempferol activate AMPK in hepatocytes through mechanisms involving SIRT1 activation and modulation of the cellular AMP:ATP ratio [131]. Both compounds reduce hepatic lipid accumulation and inflammatory signaling in experimental fatty liver disease models, and their effects are attenuated by AMPK inhibition, supporting on-target mechanisms [131,132]. Quercetin additionally activates autophagy and mitophagy in hepatocytes through AMPK–PINK1/Parkin signaling, promoting autophagic clearance of damaged mitochondria and lipid droplets, with these effects similarly abrogated by AMPK inhibition [131]. Combination of quercetin with metformin has been shown to enhance AMPK activation through the cAMP/AMPK/SIRT1 axis, further augmenting autophagic flux and reducing lipid accumulation in hepatocyte models of steatosis [132].

Curcumin, the principal bioactive polyphenol of turmeric (Curcuma longa), activates hepatic AMPK to engage the SREBP-1c/ACC-centered lipogenic suppression and β-oxidation pathway summarized in Table 1 and Section 3.3 [89]. In high-fat-diet rodent models, curcumin supplementation activates hepatic AMPK and reduces hepatic lipid accumulation and inflammatory gene expression [89,133]. Despite demonstrating potent bioactivities in preclinical settings, curcumin’s clinical application is substantially limited by its extremely poor aqueous solubility and rapid systemic elimination [134]. Honokiol, a neolignan isolated from the bark and seed cones of Magnolia species, represents an emerging natural AMPK activator with demonstrated hepatoprotective properties. Honokiol activates AMPK in hepatocytes through mechanisms that remain incompletely resolved. Early studies implicated the LKB1–AMPK axis, though more recent work suggests honokiol may act independently of classical upstream kinases (LKB1, CaMKKβ, TAK1), potentially through reported engagement of the AMPKγ1 subunit [92,135] and, at the mitochondrial level, through activation of SIRT3, a mitochondrial NAD^+^-dependent deacetylase that deacetylates and activates LKB1 [90,136]. In experimental MASLD models, honokiol reduces hepatic lipid accumulation, oxidative stress, and inflammatory signaling, and promotes lipophagy through the SIRT3–AMPK axis; these effects are substantially abolished by co-treatment with the AMPK inhibitor Compound C [91]. Honokiol further enhances mitochondrial function in lipotoxic hepatocytes, and SIRT3-dependent mitophagic mechanisms may contribute to organelle quality control [91,136].

Andrographolide, a bicyclic diterpenoid lactone from Andrographis paniculata, reduces hepatic steatosis, attenuates oxidative stress, and suppresses hepatic stellate cell activation in experimental fibrosis models [137]. Its antifibrotic effects involve inhibition of TLR4/NF-κB and TGF-β1/Smad2 signaling pathways, and reduction in hepatic stellate cell transdifferentiation [138]. In vitro co-administration studies have demonstrated that andrographolide reduces triglyceride accumulation and ROS production in steatotic hepatocytes, with evidence of differential cytoprotective and lipid-lowering contributions when combined with other polyphenols [139]. The precise upstream mechanisms through which andrographolide engages AMPK signaling in the hepatic context have not been fully delineated and warrant further mechanistic investigation.

### 4.2. Direct AMPK Activators

Recent advances in structural biology and medicinal chemistry have enabled the development of direct AMPK activators that engage the AMPK complex at defined allosteric sites, providing pharmacological activation that is independent of changes in cellular energy status. This mechanistic distinction is therapeutically significant because it allows AMPK to be activated without inducing the energetic stress associated with mitochondrial inhibitors, thereby reducing the risk of off-target energy-related adverse effects. The structural elucidation of multiple AMPK isoform complexes has provided the molecular basis for the rational design of allosteric modulators targeting distinct sites, including the ADaM (allosteric drug and metabolite) site at the interface of the kinase domain α-subunit and the carbohydrate-binding module of the β-subunit, as well as the nucleotide-binding sites on the γ-subunit [25,140].

#### 4.2.1. AICAR

5-Aminoimidazole-4-carboxamide ribonucleoside (AICAR) is a classical pharmacological activator of AMPK that functions as an AMP mimetic and has been foundational in establishing the physiological and pharmacological roles of AMPK signaling in metabolic regulation [93]. AICAR is a cell-permeable adenosine analog that is taken up via adenosine transporters and undergoes intracellular phosphorylation by adenosine kinase to form 5-aminoimidazole-4-carboxamide ribonucleoside monophosphate (ZMP), a structural analog of AMP [93,94]. ZMP binds to the CBS repeat domains (Bateman domains) on the γ-subunit of AMPK and mimics the conformational changes induced by AMP, allosterically activating the kinase and protecting Thr172 phosphorylation from phosphatase-mediated removal [93,141].

AICAR administration in animal models of metabolic disease enhances skeletal muscle glucose uptake through AMPK-dependent GLUT4 translocation, promotes fatty acid oxidation in liver and muscle, improves glucose tolerance, and reduces hepatic steatosis in diet-induced obesity models [142,143]. AICAR also suppresses inflammatory gene expression in Kupffer cells and hepatocytes through AMPK-mediated inhibition of NF-κB and STAT3 signaling pathways [144,145,146]. Despite its extensive utility in experimental AMPK research, AICAR has limited clinical applicability due to several pharmacokinetic and pharmacodynamic constraints. Its poor oral bioavailability necessitates parenteral administration, and systemic AICAR exposure leads to accumulation of ZMP in multiple tissues, which can interfere with purine biosynthesis enzymes and alter cellular nucleotide pools in ways that are independent of AMPK activation [46,94]. These off-target effects complicate the interpretation of AICAR-based studies and restrict its therapeutic development.

#### 4.2.2. A-769662

A-769662 is a direct, non-nucleotide AMPK activator [95]. The compound binds to the ADaM site formed at the interface of the β1-subunit carbohydrate-binding module (CBM) and the kinase domain of the α-subunit, a pocket that is distinct from the AMP-binding γ-subunit sites [25,147]. This allosteric drug and metabolite (ADaM) site was first structurally defined and named in the crystallographic study of Xiao et al. [25], which established the mechanistic template for all subsequent direct AMPK activators discussed in this section. A-769662 binding at this site stabilizes the phosphorylated Thr172 residue by constraining a structural conformation that protects against phosphatase access, while inducing allosteric changes that enhance intrinsic catalytic activity [95,147]. Critically, A-769662 activation of AMPK does not require changes in cellular AMP or ADP levels [95].

In preclinical metabolic disease models, A-769662 improves lipid metabolism, reduces hepatic triglyceride content, activates fatty acid oxidation, and enhances glucose homeostasis [95]. The compound shows selectivity for β1-containing AMPK complexes, which are particularly enriched in hepatocytes [96,148]. A-769662 also activates the AMPK-ULK1-autophagy axis in hepatocytes, promoting selective autophagic degradation of lipid droplets (lipophagy) and contributing to reductions in steatosis [149,150]. Despite its research utility, A-769662 exhibits poor pharmacokinetic properties, including rapid systemic clearance and limited oral bioavailability, which have precluded its clinical development [95].

#### 4.2.3. MK-8722

MK-8722 is a potent pan-AMPK activator that stimulates both β1- and β2-containing AMPK complexes by binding to the ADaM site with high affinity [68]. The compound produces robust and durable activation of AMPK across metabolic tissues including liver, skeletal muscle, and adipose tissue [68]. In short-term preclinical studies, MK-8722 produced marked improvements in glucose metabolism, reducing fasting plasma glucose, improving glucose tolerance, and enhancing insulin sensitivity in diet-induced obese and genetic models of insulin resistance [68]. These beneficial metabolic effects were accompanied by reduced hepatic lipid content and improvements in plasma lipid profiles [12,68]. However, longer-term administration of MK-8722 was associated with the development of cardiac hypertrophy and glycogen accumulation in cardiomyocytes [68]. These cardiac effects are mechanistically attributed to activation of β2-containing AMPK complexes in cardiac tissue, which stimulate glucose and fatty acid uptake and promote anabolic glycogen synthesis pathways in the myocardium [68,99].

#### 4.2.4. PF-06409577

Building on the structural insights provided by A-769662 [95,151], a series of highly potent, β1-selective AMPK activators with substantially improved pharmacokinetic profiles have been developed [97,152]. PF-06409577 is an indole-based compound that binds the ADaM site with high affinity and selectivity for β1-containing AMPK complexes [64,97,153]. This isoform selectivity is particularly advantageous because it allows hepatic AMPK signaling to be engaged at doses that do not substantially activate β2-enriched complexes in skeletal muscle and cardiac tissue, potentially mitigating adverse effects associated with systemic pan-AMPK activation [68,152].

Treatment with PF-06409577 in rodent models of diet-induced obesity and insulin resistance significantly reduced hepatic lipid accumulation via the FASN/ACC1-suppressing mechanism outlined in Table 1, and additionally improved insulin sensitivity and reduced plasma triglyceride levels [64]. The compound also activated hepatic autophagy and mitophagy, improved mitochondrial quality control, and reduced hepatocyte apoptosis in models of lipotoxic injury [12,64]. Importantly, PF-06409577 did not induce cardiac hypertrophy or adversely affect skeletal muscle function at therapeutically relevant doses [64,153]. A related compound in this structural series, PF-249 (also designated Compound 44), demonstrated oral bioavailability sufficient for once-daily dosing and produced durable improvements in hepatic steatosis and fibrosis markers in preclinical MASLD models [64,153]. Despite this preclinical promise, clinical development of PF-06409577 itself does not appear to have advanced toward a liver indication: its only registered trial, a first-in-human single-ascending-dose study in healthy volunteers conducted for the proposed diabetic nephropathy indication, was terminated in 2015 for business rather than safety reasons, and no subsequent trials of PF-06409577, PF-249, or Compound 991—whether for diabetic nephropathy, other metabolic conditions, or MASLD/MASH—appear to have been registered (NCT02286882). The ADaM site, β1-selective direct-activator class therefore remains at the preclinical stage of translation for liver disease, and dedicated MASLD/MASH-focused clinical trials, rather than reliance on repurposed diabetes- or nephropathy-oriented programs, will be required to establish genuine clinical potential in this indication.

#### 4.2.5. Compound 991

Compound 991 (also designated Ex229) is a potent ADaM site activator that demonstrates high selectivity for β1-containing AMPK complexes. Compound 991 activates AMPK with greater potency than A-769662 and exhibits markedly improved metabolic stability in microsomal assays [25,98]. In primary hepatocytes and in vivo rodent studies, Compound 991 robustly suppresses hepatic lipogenesis through the ACC-phosphorylation mechanism summarized in Table 1 and additionally reduces mTORC1 activity and activates autophagy [37,98].

#### 4.2.6. O304

O304 is an orally available pan-AMPK activator. In contrast to ADaM site activators such as MK-8722, which allosterically enhance AMPK catalytic activity by binding between the α- and β-subunits, O304 is a first-in-class compound with a mechanistically distinct profile. It increases AMPK activity by suppressing PP2C-mediated dephosphorylation of pAMPK at Thr172, thereby sustaining the active phosphorylated state rather than directly enhancing intrinsic catalytic rate [99]. The precise binding site of O304 within the AMPK heterotrimer has not yet been fully characterized [99]. In preclinical studies, O304 improved insulin sensitivity, reduced fasting plasma glucose, and protected pancreatic β-cell function in diet-induced obese mouse models [99,100]. O304 also improved cardiac function and exercise capacity in aged mice, effects the authors attributed to exercise-mimetic physiological cardiac adaptation rather than pathological remodeling [101]. Notably, despite being a pan-AMPK activator, O304 did not induce pathological cardiac hypertrophy or glycogen accumulation in animal models at therapeutic doses [99]. O304 has completed Phase I and Phase IIa clinical trials in type 2 diabetes patients, where it demonstrated statistically significant reductions in fasting plasma glucose and HOMA-IR, and improvements in peripheral microvascular perfusion and blood pressure, with an acceptable safety and tolerability profile [99,100]. Notably, these trials enrolled patients with type 2 diabetes rather than MASLD or MASH, and to our knowledge no registered clinical trial has yet evaluated O304 (recently redesignated ATX-304) specifically in a MASLD/MASH population. The rationale for such a trial has strengthened; however, in a preclinical choline-deficient high-fat-diet mouse model of progressive MASLD, ATX-304 reduced whole-body fat mass, hepatic steatosis, oxidative lipid stress, and fibrosis progression while inducing a hepatic metabolic shift toward fatty acid oxidation [154]. Translating this preclinical hepatic benefit into a dedicated MASLD/MASH clinical trial—prioritizing liver-focused endpoints such as MRI-derived proton density fat fraction, histological fibrosis staging, or noninvasive fibrosis biomarkers, rather than the glycemic and microvascular endpoints used in the completed T2D trials—represents an important and currently unmet opportunity for this compound.

#### 4.2.7. ZLN024

ZLN024 is a direct AMPK activator. ZLN024 activates AMPK in hepatocytes by binding to the ADaM site and has demonstrated potent suppression of hepatic lipogenesis in experimental models [103], based on allosteric activation of all four AMPK heterotrimer combinations (α1β1γ1, α2β1γ1, α1β2γ1, and α2β2γ1), with EC50 values of 0.42, 0.95, 1.1, and 0.13 μM, respectively; unlike A-769662, PF-06409577, and Compound 991, ZLN024 therefore lacks pronounced β1-selectivity, instead activating β2-containing complexes with comparable or even greater potency than β1-containing complexes. As with A-769662, ZLN024 requires pre-phosphorylation of AMPKα Thr172 by an upstream kinase and subsequently protects this residue from dephosphorylation by PP2Cα, without altering the cellular ADP/ATP ratio, consistent with a genuinely allosteric mechanism rather than indirect activation through energy-charge perturbation. In db/db mice, ZLN024 (15 mg/kg/day) improved glucose tolerance and reduced liver weight and hepatic triglyceride and cholesterol content, accompanied by reduced hepatic expression of the gluconeogenic and lipogenic genes G6Pase, FASN, and mtGPAT, increased skeletal muscle expression of fatty acid oxidation and mitochondrial biogenesis genes, and enhanced ACC phosphorylation in both liver and muscle. To our knowledge, no additional binding-mode, selectivity, or in vivo efficacy studies of ZLN024 have been published since this original report, leaving structural confirmation of its ADaM site interaction and its efficacy in dedicated hepatic fibrosis models as open questions for future work. Broader antifibrotic effects of AMPK activation in hepatic stellate cells, including suppression of TGF-β-driven Smad2/3 signaling, have been described, suggesting that direct AMPK activators may have the capacity to simultaneously address multiple pathological drivers of MASLD progression (steatosis, inflammation, and fibrosis) within a single pharmacological agent [62,155].

## 5. Challenges in Clinical Translation of AMPK-Targeting Strategies

The clinical translation of AMPK-targeting strategies has proven considerably more difficult than originally anticipated. The biological ubiquity of AMPK, the structural heterogeneity of its constituent isoforms, the pharmacokinetic limitations of many candidate compounds, and the multifactorial nature of MASLD itself collectively impose formidable barriers at each stage of translational development [47,156]. These challenges delineate the specific scientific and pharmacological problems that must be resolved before AMPK activators can be advanced into routine clinical practice. Understanding these limitations is a prerequisite for informed drug development.

### 5.1. Systemic Effects

One of the most consequential obstacles to the clinical deployment of AMPK activators is the ubiquitous expression of AMPK across virtually all mammalian tissues [157]. While hepatic AMPK activation is desirable for its capacity to suppress lipid synthesis, enhance fatty acid oxidation, and reduce hepatic glucose output, the same kinase fulfills markedly different and sometimes opposing physiological roles in other organ systems. In skeletal muscle, AMPK activation promotes glucose uptake and oxidative metabolism, effects that are generally considered beneficial in the context of insulin resistance [158,159]. However, in the hypothalamus, AMPK acts as an orexigenic signal; its activation has been shown in preclinical models to stimulate appetite and increase food intake, an outcome that would be counterproductive in a population already predisposed to caloric excess and obesity [160,161]. In adipose tissue, AMPK exerts inhibitory effects on lipolysis under certain conditions, potentially reducing the mobilization of free fatty acids to the liver and thus blunting the very substrate overflow that contributes to hepatic lipid accumulation in MASLD [67,162]. Yet paradoxically, chronic systemic AMPK activation could disrupt the delicate hormonal and metabolic crosstalk between adipose tissue and the liver. Cardiac AMPK, too, plays an indispensable role in regulating myocardial energy balance during ischemia; nonselective perturbation of this pathway risks interfering with an adaptive cardioprotective mechanism [163,164]. The cumulative consequence of these tissue-level divergences is that a systemically administered AMPK activator may yield a net metabolic signal that is beneficial in one organ while simultaneously producing unintended or adverse consequences in another. This tissue-wide pharmacodynamic complexity substantially complicates both the preclinical evaluation and the clinical risk–benefit calculus of such agents [47,156,157].

### 5.2. Isoform Selectivity

Closely related to the issue of systemic effects is the challenge of isoform selectivity. AMPK is a heterotrimeric enzyme composed of a catalytic α-subunit and regulatory β- and γ-subunits, each of which exists in multiple isoforms (α1, α2; β1, β2; γ1, γ2, γ3). The combinatorial diversity of these subunits permits the formation of up to twelve distinct AMPK heterotrimers, whose expression patterns, subcellular localizations, and functional specificities vary considerably among tissues [20,165]. The α1-containing complexes, for example, are broadly expressed and tend to predominate in lipogenic tissues, whereas α2-containing complexes are more prevalent in skeletal muscle and heart, where they are preferentially activated during energetic stress [165,166]. The β1 isoform is highly expressed in the liver and has been identified as a critical determinant of hepatic lipid metabolism, making it a particularly attractive target for MASLD pharmacotherapy [47,167].

Most first-generation AMPK activators, including the widely studied indirect activator metformin and the direct small-molecule activator AICAR, exhibit little to no isoform selectivity [168]. This pharmacological bluntness limits their utility and confounds the interpretation of preclinical and clinical outcomes, since the observed effects may reflect the composite activation of multiple AMPK complexes with disparate downstream functions. The development of isoform-selective activators, such as the β1-selective compound A-769662 and its structural descendants, has provided proof-of-concept evidence that tissue-enriched pharmacology is achievable, but translating this selectivity from the bench to a clinically viable molecule remains technically demanding [95,167].

### 5.3. Pharmacokinetics

Even when a compound demonstrates robust AMPK activation and acceptable selectivity in vitro, the translation of these properties into a clinically effective drug is frequently undermined by unfavorable pharmacokinetic profiles. Oral bioavailability is perhaps the most commonly encountered limitation: many AMPK-activating molecules, particularly those derived from natural product scaffolds such as berberine or resveratrol, are subject to extensive first-pass hepatic metabolism or poor intestinal absorption, resulting in systemic exposures that are far below the concentrations required for meaningful target engagement [169,170,171,172]. Beyond bioavailability, the metabolic stability of AMPK activators is a recurring concern. Rapid degradation by hepatic cytochrome P450 enzymes or phase II conjugation reactions shortens plasma half-lives, necessitating frequent dosing regimens that may diminish patient adherence, where long-term compliance is essential for meaningful histological benefit [173,174]. Drug–drug interaction potential, volume of distribution, and the ability to achieve adequate intracellular concentrations at the hepatocyte level are additional pharmacokinetic parameters that must be optimized during lead compound refinement. Novel delivery strategies, including nanoparticle-based formulations, lipid carriers, and targeted prodrug approaches, are being investigated to circumvent some of these limitations, but none has yet achieved clinical validation in the MASLD context [175,176].

### 5.4. Disease Complexity

Perhaps the most formidable challenge confronting the clinical translation of AMPK activators is the intrinsic biological complexity of MASLD itself. The disease encompasses a continuous histopathological spectrum, from simple hepatic steatosis through MASH to progressive fibrosis, cirrhosis, and hepatocellular carcinoma, and its natural history is shaped by an extraordinarily heterogeneous interplay of metabolic, inflammatory, fibrogenic, and genetic factors [177,178]. Lipid accumulation in the hepatocyte is the initiating event, but it is the subsequent cascade of lipotoxicity, oxidative stress, mitochondrial dysfunction, endoplasmic reticulum stress, and innate immune activation that drives the transition from simple steatosis to overt hepatocellular injury [179,180]. AMPK, though a powerful regulator of hepatic lipid metabolism, is only one node within this expansive pathophysiological network. Its activation can ameliorate steatosis and attenuate some aspects of metabolic inflammation, but it does not directly address the activated hepatic stellate cells that drive fibrogenesis, nor does it fully counteract the inflammatory cytokine milieu generated by Kupffer cell activation and gut-derived endotoxin signaling [181,182]. Monotherapy with an AMPK activator may yield measurable reductions in hepatic fat content, while leaving fibrosis scores largely unchanged [183]. Furthermore, the heterogeneity of the MASLD patient population, with respect to both disease stage and underlying metabolic comorbidities, complicates the identification of the patient subgroups most likely to benefit from AMPK-centric interventions. A patient in the early steatotic phase driven primarily by insulin resistance and de novo lipogenesis may respond very differently from one with established fibro-inflammatory disease, where the dominant pathological processes are largely downstream of the metabolic derangements that AMPK corrects [184]. These realities have prompted increasing interest in combination therapeutic strategies that pair AMPK activation with agents targeting complementary disease mechanisms—most notably FXR agonists to improve bile acid homeostasis, FGF21 analogs to modulate adipose tissue metabolism, ACC inhibitors to directly suppress de novo lipogenesis, or antifibrotic agents such as CCR2/CCR5 antagonists [185,186,187,188].

Direct evidence testing an AMPK activator together with an FXR agonist in a rodent model of MASLD is, to our knowledge, not yet available; PF-06409577 reduces markers of hepatic fibrosis as a monotherapy in an AMPK-dependent manner [64], but this compound class has not been formally combined with an FXR agonist, representing an opportunity for future preclinical work rather than an established finding. The rationale for such pairing is nonetheless reinforced by related preclinical data showing that AMPK activator monotherapy consistently uncouples metabolic and steatotic benefit from histological fibrosis regression: systemic activation with the β1/β2 AMPK activator BI9774 markedly reduced hepatic steatosis, plasma transaminases, and collagen transcripts in Lep-ob/Lep-ob (ob/ob) MASH mice, yet collagen protein deposition and overall histological fibrosis were unchanged after six weeks of treatment [63], whereas the β1-selective activator PXL770 achieved measurable reductions in hepatic inflammation and fibrogenesis in a comparable model only after an extended eight-week treatment course [189]. These findings support the premise that a complementary agent may be needed to translate AMPK-driven metabolic improvement into fibrosis regression within a clinically feasible timeframe. Among the pairings noted above, combining a CCR2/CCR5 antagonist with a metabolically active agent has the most direct preclinical support: a novel oral CCR2/CCR5 antagonist (BMS-687681, mechanistically related to but distinct from the clinically tested antagonist cenicriviroc) combined with a pegylated FGF21 agonist (BMS-986171) produced synergistic improvements in steatohepatitis activity and fibrosis stage compared with either agent alone in a preclinical MASH model [188]. A comparable principle applies to FXR-directed combinations: because activated hepatic stellate cells become relatively resistant to FXR agonism, pairing the FXR agonist obeticholic acid with agents that restore stalled FXR signaling in activated HSCs markedly enhanced antifibrotic efficacy over obeticholic acid alone in mouse models of liver fibrosis [190], illustrating a mechanism by which an AMPK activator, which independently suppresses HSC activation, could plausibly potentiate FXR agonist efficacy. Not every mechanistically plausible combination has translated into added antifibrotic benefit; however, pairing an ACC inhibitor with several peroxisome proliferator-activated receptor or thyroid hormone receptor-β agonists prevented ACC-inhibitor-induced hypertriglyceridemia in dyslipidemic rats, but only a subset of these combinations provided additional benefit on fibrosis endpoints beyond ACC inhibition alone [191]. Together, these examples indicate that combining AMPK activators with FXR agonists, FGF21 analogs, ACC inhibitors, or CCR2/CCR5 antagonists is mechanistically well-justified and, in analogous drug classes, capable of yielding true synergy, but that dedicated empirical testing, rather than assumed additivity, will be required to identify which specific pairings deliver meaningful gains in fibrosis outcomes.

## 6. Pharmaceutical Approaches to Enhance the Druggability of AMPK Activators

Plant-derived indirect activators such as curcumin, berberine, and resveratrol combine low aqueous solubility with extensive presystemic metabolism, yielding low oral bioavailabilities. First-generation direct activators, in turn, have been constrained by poor oral absorption, incomplete isoform selectivity, and, in the case of systemic pan-activators, on-target cardiac liabilities. Three complementary pharmaceutical strategies have been adopted to close this druggability gap: (1) formulation- and delivery-based enhancement of bioavailability, (2) prodrug design, and (3) structure-guided optimization of potency, isoform selectivity, and tissue distribution.

### 6.1. Bioavailability Enhancement of Natural (Indirect) Activators

Recent pharmaceutical innovations, including phospholipid complexes (phytosomes), self-emulsifying drug delivery systems, and nanoparticle formulations, have improved systemic curcumin exposure. The nanoparticle strategies span three mechanistically distinct carrier classes: surfactant-based nanomicelles, polymeric nanoparticles, and lipid-matrix systems. Nanomicellar curcumin (e.g., SinaCurcumin) has been the formulation actually carried into NAFLD trials, where its improved solubility and absorption contribute to the reductions in hepatic steatosis and aminotransferases observed clinically [192,193]. Among polymeric carriers, poly(lactic-co-glycolic acid) (PLGA) nanoparticles have been reported to increase curcumin bioavailability roughly 22-fold, while curcumin-loaded solid lipid nanoparticles (~150 nm) reduced serum ALT, AST, TNF-α, and oxidative stress in the CCl_4_-injured liver, indicating that the lipid matrix protects the labile diketone moiety from premature metabolism while favoring hepatic delivery [194,195]. These trials are small and short-term, but they establish a proof of concept that solubility- and absorption-directed formulation can render a metabolically labile polyphenol clinically active.

In case of berberine, despite consistent glucose- and lipid-lowering activity in type 2 diabetes and NAFLD, its oral bioavailability is below 1%, reflecting poor aqueous solubility, P-glycoprotein-mediated intestinal efflux, and extensive first-pass metabolism. Advanced delivery systems have addressed each barrier with different carrier systems. The chitosan–alginate system prepared by ionic gelation and optimized by a Box–Behnken design yielded 202 nm particles (PDI 0.24, zeta −14.8 mV) with 86% entrapment efficiency, and improved both ex vivo intestinal permeation and in vivo oral bioavailability [196]; the cationic chitosan shell contributes mucoadhesion and transient tight-junction opening, helping circumvent P-glycoprotein efflux. Solid lipid nanoparticles (SLNs) have driven berberine particle size below 80 nm with improved bioavailability, and chitosan-coated SLN hybrids combine lipid-mediated lymphatic uptake—which partially bypasses hepatic first-pass metabolism—with mucoadhesive surface chemistry [197,198]. Because lipid-based carriers can additionally favor hepatic accumulation, they direct the drug toward the principal site of berberine’s metabolic action. Medicinal-chemistry approaches are complementary: dihydroberberine, a reduced analog that is absorbed more efficiently and re-oxidized to berberine in vivo, achieves substantially higher plasma exposure than the parent compound at equivalent doses.

Resveratrol illustrates an important limitation of formulation-only strategies. Although it is reasonably well absorbed, near-complete first-pass glucuronidation and sulfation, together with enterohepatic recirculation, leave very low concentrations of the parent compound in the systemic circulation. The micronized formulation SRT501 increased mean plasma resveratrol roughly 3.6-fold relative to equivalent doses of unformulated drug in a clinical pharmacokinetic study, demonstrating that particle-size reduction can improve absorption [199]. The instructive contrast is between simple particle-size reduction and carriers engineered against the metabolic barrier itself. Nanocarriers (polymeric nanoparticles, SLNs, liposomes, and micelles) can enhance resveratrol exposure not only by improving dissolution but by modulating P-glycoprotein and cytochrome P-450 activity and routing drug through intestinal lymphatic transport to bypass the hepatic first-pass effect. Resveratrol-loaded SLN, for instance, roughly doubled tissue bioavailability in liver, kidney, and brain versus free drug. The most direct test of this logic is receptor-targeted hepatic delivery: galactose-modified lipid nanoparticles exploiting asialoglycoprotein-receptor-mediated endocytosis raised intracellular resveratrol uptake 3.5-fold and, in NAFLD mice, lowered hepatic lipid accumulation and reduced serum ALT and AST by roughly 48% and 59%/49%, respectively, outperforming free resveratrol [200].

### 6.2. Prodrug Strategies: Salsalate and Salicylate

Prodrug design addresses a different facet of druggability–tolerability and the duration of exposure–rather than solubility. Salicylate is one of the few direct AMPK activators with a long clinical history: it binds the β1-subunit allosteric drug and metabolite (ADaM) site, the same pocket engaged by the synthetic activator A-769662, producing allosteric activation and protecting Thr172 from dephosphorylation [66,95]. Attaining the plasma salicylate concentrations required for AMPK activation (approximately 1 mmol/L) with salicylate or aspirin is limited by gastrointestinal tolerability. Salsalate, a dimeric prodrug hydrolyzed in the small intestine to two molecules of salicylate, provides more sustained salicylate exposure with improved tolerability and lowers blood glucose in type 2 diabetes while reducing hepatic lipid content in NAFLD models [201]. Notably, several of salsalate’s metabolic benefits—including mitochondrial uncoupling—are AMPK-independent, so the prodrug enhances the druggability of a multi-mechanistic agent rather than of a pure AMPK activator [201].

### 6.3. Medicinal-Chemistry Optimization of Direct Synthetic Activators

The trajectory of direct allosteric activators shows how medicinal chemistry has progressively converted research tools into candidate drugs. A-769662, a β1-selective thienopyridone, validated the pharmacology of direct ADaM site activation but suffered from poor oral bioavailability that largely confined it to experimental use [95]. The PF-06409577 was engineered specifically to possess high potency and selectivity for β1-containing complexes (EC50 ≈ 7 nM for α1β1γ1 versus negligible activity at β2 complexes). It gained oral absorption through a deliberate scaffold change from an indazole to an indole core, improving both permeability and solubility, and advanced to first-in-human trials for diabetic nephropathy [97]. Because β1-containing heterotrimers predominate in liver and kidney, β1-selectivity also confers a degree of tissue-directed action. The PF-06409577 improved kidney function in a rat model of diabetic nephropathy [153] and corrected NAFLD while lowering cholesterol in rodent and non-human primate models [64].

The contrasting profiles of two systemic pan-activators illustrate the central safety challenge of nonselective direct activation. MK-8722, which activates all twelve αβγ heterotrimer combinations, produced potent and durable glucose-lowering across rodent and non-human primate models but induced cardiac hypertrophy associated with glycogen accumulation, an on-target consequence of indiscriminate AMPK activation in the heart [68]. By comparison, the pan-activator O304, which sustains AMPK activation by inhibiting Thr172 dephosphorylation and has been administered to humans, reduced fasting glucose and HOMA-IR in a Phase IIa study in metformin-treated type 2 diabetes patients and improved cardiac functional measures without increasing heart weight in preclinical studies [202].

Beyond the heart, the long-term consequences of chronic pharmacological AMPK activation in other organs remain incompletely defined and warrant a cautious interpretation of the safety data reviewed above. In the kidney, AMPK contributes to the regulation of tubular sodium transport, including modulation of the epithelial sodium channel and Na^+^/K^+^-ATPase activity, so sustained pharmacological activation could in principle alter renal sodium handling and fluid balance over time, an effect that has not been systematically characterized in chronic dosing studies of direct AMPK activators. In the central nervous system, hypothalamic AMPK activity is a key regulator of appetite and energy expenditure, and agents capable of engaging central AMPK could conceivably perturb feeding behavior or body-weight regulation in ways not predicted by peripheral metabolic studies alone. Because preclinical evaluation of AMPK activators has focused predominantly on hepatic, metabolic, and cardiac endpoints, dedicated long-term toxicology and clinical monitoring of renal and central nervous system effects will be important for a fully balanced safety assessment of these agents.

## 7. Conclusions

AMPK occupies a central position in the pathophysiology and therapeutic landscape of MASLD. As a main regulator of hepatic energy homeostasis, AMPK coordinates the metabolic processes that are disrupted in the disease, including lipogenesis, fatty acid oxidation, gluconeogenesis, cholesterol synthesis, and inflammatory and fibrogenic signaling. Chronic suppression of hepatic AMPK, driven by hyperinsulinemia, lipid intermediates, oxidative stress, and inflammatory cues, contributes directly to disease progression, making restoration of AMPK activity a rational therapeutic strategy capable of simultaneously targeting metabolic dysfunction, inflammation, and fibrogenesis. Pharmacological approaches to AMPK activation have expanded from established indirect activators such as metformin and berberine to a new generation of direct allosteric activators that engage defined regulatory sites within the AMPK complex. Structural insights from crystallography and cryo-electron microscopy have enabled increasingly selective compounds, including β1-preferring activators such as PF-06409577 and Compound 991, while newer agents such as O304 demonstrate that distinct activation mechanisms may offer improved safety profiles. Despite this progress, important translational challenges remain. Because AMPK is expressed across multiple tissues with divergent physiological roles, effective therapy will likely require liver-selective strategies, including isoform-targeted activators, hepatocyte-directed delivery systems, or prodrug approaches. Of these two directions, we consider isoform-selective (β1-biased) small molecules more likely to reach clinical evaluation within the next five years, since they build on medicinal-chemistry platforms and oral pharmacokinetic profiles already validated in humans, such as PF-06409577, whereas hepatocyte-directed nanocarrier and prodrug delivery systems, although conceptually well suited to restricting AMPK activation to the liver, still face substantial manufacturing, regulatory, and long-term safety hurdles that will likely place their clinical translation on a longer horizon. In addition, the heterogeneous and stage-dependent nature of MASLD necessitates patient stratification and precision-medicine frameworks to identify individuals most likely to benefit from AMPK-centered interventions. Candidate biomarkers for such stratification include hepatic AMPK activity or phosphorylation status, ideally captured through noninvasive surrogates rather than biopsy; functional genetic variants in PRKAA1/PRKAA2 that may influence baseline AMPK activity or pharmacological responsiveness; and circulating metabolic markers of AMPK pathway engagement, such as FGF21, adiponectin, or specific lipid and acylcarnitine species; however, systematic clinical validation of these candidates for predicting AMPK activator response in MASLD is still lacking, and their prospective incorporation into future trial designs represents an important direction for precision medicine in this field. Finally, AMPK activation alone may not fully address advanced disease, particularly established fibrosis. Combination strategies, pairing AMPK activators with agents targeting complementary pathways such as FXR, FGF21, ACC, or antifibrotic signaling, may therefore provide the most effective therapeutic framework, although direct preclinical or clinical evidence for several of these specific pairings involving AMPK activators remains limited and represents an important direction for future study. With continued advances in structural biology, medicinal chemistry, and disease stratification, AMPK remains one of the most promising and mechanistically coherent targets in MASLD drug development.

## Figures and Tables

**Table 1:** Pharmacological activators of AMPK relevant to MASLD therapy. The ACC/SREBP-1c/HMGCR/PGC-1α mechanistic detail underlying the “Key Metabolic Effects” column is described once in Section 3.3; Section 4 refers back to this table rather than restating that mechanistic detail for each compound.

Compound	Primary Mechanism of AMPK Activation	Direct vs. Indirect	Key Metabolic Effects Relevant to MASLD	Refs.
Metformin	Inhibits mitochondrial respiratory chain complex I → increases AMP/ATP ratio → indirect AMPK activation	Indirect	Suppresses hepatic gluconeogenesis; inhibits lipogenesis via ACC and SREBP-1c inhibition; promotes fatty acid oxidation	[16,76,77]
Berberine	Inhibits mitochondrial complex I → increases cellular AMP/ADP → AMPK activation	Indirect	Reduces hepatic lipid accumulation; improves insulin sensitivity; suppresses inflammatory signaling	[78,79,80]
Resveratrol	Activates AMPK through SIRT1–LKB1 signaling and phosphodiesterase inhibition → increased cAMP/Epac signaling	Indirect	Enhances mitochondrial biogenesis and fatty acid oxidation; promotes autophagy and lipophagy	[81,82,83]
EGCG (green tea catechin)	Indirect AMPK activation associated with LKB1/SIRT1 signaling and mild mitochondrial perturbation	Indirect	Reduces hepatic steatosis; enhances fatty acid oxidation and autophagy	[84,85]
Quercetin/Kaempferol	Modulate AMP/ATP ratio and activate SIRT1–AMPK signaling	Indirect	Reduce hepatic lipid accumulation and inflammatory signaling	[86,87]
Curcumin	Activates LKB1-AMPK signaling in hepatocytes; promotes p-AMPK-mediated inhibition of ACC and SREBP-1c	Indirect	Suppresses de novo lipogenesis and enhances fatty acid oxidation; reduces hepatic steatosis in NAFLD models	[88,89]
Honokiol	Activates SIRT3–AMPK signaling axis in hepatocytes; also reported to engage the AMPKγ1 subunit in NAFLD models	Indirect (with reported direct component)	Reduces hepatic steatosis and oxidative stress; promotes SIRT3-AMPK-mediated lipophagy in hepatocytes	[90,91,92]
AICAR (ZMP precursor)	Converted intracellularly to ZMP, an AMP mimetic that binds γ-subunit CBS domains and activates AMPK	Direct (via active metabolite ZMP)	Enhances fatty acid oxidation and glucose uptake; reduces hepatic steatosis in experimental models	[93,94]
A-769662	Direct binding to ADaM site at α-β interface stabilizing Thr172 phosphorylation; β1-selective	Direct	Suppresses lipogenesis and increases fatty acid oxidation	[95,96]
PF-06409577 ^a^	β1-selective ADaM site activator preferentially targeting hepatic AMPK complexes	Direct	Reduces hepatic lipid synthesis, lowers cholesterol and markers of fibrosis in preclinical models	[64,97]
MK-8722	Potent pan-AMPK ADaM site activator activating multiple AMPK heterotrimers	Direct	Improves glucose tolerance and lipid metabolism	[68]
Compound 991 (Ex229)	Potent β1-biased ADaM site activator; 5–10× more potent than A-769662; activates β1- and β2-containing complexes	Direct	Suppresses lipogenesis and activates autophagy; inhibits lipogenesis in hepatocytes at low nanomolar concentrations	[25,98]
O304	Suppresses PP2C-mediated dephosphorylation of pAMPK at Thr172, sustaining the active phosphorylated state independent of ADaM site binding	Indirect (phosphatase-inhibition mechanism, non-ADaM)	Improves insulin sensitivity and reduces fasting glucose; favorable cardiac safety profile; completed Phase IIa trials in type 2 diabetes	[99,100,101]
ZLN024	Direct AMPK activator identified through cell-based screening	Direct	Suppresses hepatic lipogenesis and metabolic dysfunction	[102,103]

^a^ PF-249 (Compound 44) is a structurally related analog in the same PF-06409577 chemical series, sharing its ADaM site, β1-selective mechanism; see Section 4.2.4 for its distinct preclinical dataset in MASLD models.

## Data Availability

No new data were created or analyzed in this study. Data sharing is not applicable to this article.

## Abbreviations

ACC	Acetyl-CoA carboxylase
AICAR	5-Aminoimidazole-4-carboxamide ribonucleotide
AID	Autoinhibitory domain
ADaM	Allosteric drug and metabolite (site)
AMPK	AMP-activated protein kinase
AS160	Akt substrate of 160 kDa
AST/ALT	Aspartate aminotransferase/alanine aminotransferase
ATP/ADP/AMP	Adenosine triphosphate/diphosphate/monophosphate
CaMKKβ	Calcium/calmodulin-dependent protein kinase kinase β
CBM/GBD	Carbohydrate-binding module/glycogen-binding domain
CBS	Cystathionine-β-synthase
CCR2/CCR5	C-C chemokine receptor type 2/type 5
CD4/CD8	Cluster of differentiation 4/8
ChREBP	Carbohydrate response element-binding protein
CPT1	Carnitine palmitoyltransferase-1
CRTC2/TORC2	CREB-regulated transcription coactivator 2
CTD	C-terminal domain
ECM	Extracellular matrix
EGCG	Epigallocatechin-3-gallate
FASN	Fatty acid synthase
FDA	US Food and Drug Administration
FGF21	Fibroblast growth factor 21
FOXO	Forkhead box O
FXR	Farnesoid X receptor
GLUT1/GLUT4	Glucose transporter 1/4
HDAC4	Histone deacetylase 4
HIF	Hypoxia-inducible factor
HMGCR	3-Hydroxy-3-methylglutaryl-CoA reductase
HOMA-IR	Homeostatic model assessment of insulin resistance
HSC	Hepatic stellate cell
ILC1/ILC3	Innate lymphoid cell type 1/type 3
KD	Kinase domain
LKB1	Liver kinase B1
LPS	Lipopolysaccharide
MASH	Metabolic dysfunction-associated steatohepatitis
MASLD	Metabolic dysfunction-associated steatotic liver disease
mGPD	Mitochondrial glycerophosphate dehydrogenase
MMP	Matrix metalloproteinase
MO25	Mouse protein 25
MRI	Magnetic resonance imaging
mTORC1	Mechanistic target of rapamycin complex 1
NAFLD	Nonalcoholic fatty liver disease
NASH	Nonalcoholic steatohepatitis
NF-κB	Nuclear factor-κB
NLRP3	NOD-, LRR- and pyrin domain-containing protein 3
OCT1	Organic cation transporter 1
PDI	Polydispersity index
PEPCK	Phosphoenolpyruvate carboxykinase
PFKFB3	6-Phosphofructo-2-kinase/fructose-2,6-biphosphatase 3
PGC-1α	Peroxisome proliferator-activated receptor-γ coactivator 1-alpha
PI3K	Phosphoinositide 3-kinase
PINK1	PTEN-induced kinase 1
PLGA	Poly(lactic-co-glycolic acid)
PP2A/PP2C	Protein phosphatase 2A/2C
PPARα	Peroxisome proliferator-activated receptor alpha
PRKAA1/PRKAA2	Genes encoding AMPK catalytic subunits α1/α2
PRKAB1/PRKAB2	Genes encoding AMPK regulatory subunits β1/β2
ROS	Reactive oxygen species
SCD1	Stearoyl-CoA desaturase 1
SIRT1/SIRT3	Sirtuin 1/sirtuin 3
SLNs	Solid lipid nanoparticles
SREBP-1c	Sterol regulatory element-binding protein-1c
STAT3	Signal transducer and activator of transcription 3
STING	Stimulator of interferon genes
STK11	Serine/threonine kinase 11
STRADα/β	STE20-related kinase adaptor alpha/beta
T2D	Type 2 diabetes
TAK1	TGF-β-activated kinase 1
TBC1D1/TBC1D4	TBC1 domain family member 1/4
TGF-β	Transforming growth factor beta
TIMP-1	Tissue inhibitor of metalloproteinases-1
TLR4	Toll-like receptor 4
TNF-α	Tumor necrosis factor alpha
Treg/Th17	Regulatory T cell/T helper 17 cell
TSC2	Tuberous sclerosis complex 2
TXNIP	Thioredoxin-interacting protein
ULK1	Unc-51-like autophagy-activating kinase 1
ZMP	5-Aminoimidazole-4-carboxamide ribonucleotide monophosphate
